# Age Effects on Controlling Tools with Sensorimotor Transformations

**DOI:** 10.3389/fpsyg.2012.00573

**Published:** 2012-12-24

**Authors:** Christine Sutter, Stefan Ladwig, Michael Oehl, Jochen Müsseler

**Affiliations:** ^1^Work and Cognitive Psychology, Institute of Psychology, RWTH Aachen UniversityAachen, Germany; ^2^Institute of Experimental Industrial Psychology, Leuphana University of LüneburgLüneburg, Germany

**Keywords:** distal action effect, ideomotor principle, perception, proprioception, proximal action effect, sensory integration, tool use, vision

## Abstract

Controlling tools in technical environments bears a lot of challenges for the human information processing system, as locations of tool manipulation and effect appearance are spatially separated, and distal action effects are often not generated in a 1:1 manner. In this study we investigated the susceptibility of older adults to distal action effects. Younger and older participants performed a Fitts’ task on a digitizer tablet without seeing their hand and the tablet directly. Visual feedback was presented on a display in that way, that cursor amplitude and visual target size varied while the pre-determined hand amplitude remained constant. In accordance with distal action effects being predominant in controlling tool actions we found an increase in hand movement times and perceptual errors as a function of visual task characteristics. Middle-aged adults more intensely relied on visual feedback than younger adults. Age-related differences in speed-accuracy trade-off are not likely to account for this finding. However, it is well known that proprioceptive acuity declines with age. This might be one reason for middle-aged adults to stronger rely on the visual information instead of the proprioceptive information. Consequently, design and application of tools for elderly should account for this.

## Introduction

Controlling tools in technical environments bears a lot of challenges for the human information processing system, as locations of tool manipulation and effect appearance are spatially separated, and distal effects are often not generated in a 1:1 manner. When processing discordant information – for instance – from the moving hand (proximal action effect) and a moving cursor on a monitor (distal action effect) diverse crosstalk between proximal and distal action effects affect short-term compensation for as well as long-term adaptation to changes in sensorimotor transformations (e.g., Rieger et al., 2005; Heuer and Hegele, 2009; Ladwig et al., 2012; Sülzenbrück and Heuer, 2012; for recent overviews of empirical evidence see, e.g., Sutter and Sülzenbrück, 2012; Sutter et al., in press). Ladwig et al. (2012) let participants perform hand movements on a covered digitizer tablet while different gain factors varied the cursor amplitude, so that the cursor amplitude was shorter, equal or longer than the hand amplitude. Participants had to replicate the formerly performed hand amplitude as exactly as possible (but now without visual feedback) after finishing a trial. Drawn hand amplitudes were very exact when hand and cursor amplitude of the former movement corresponded. But, they were shorter (longer) when the former cursor amplitude was shorter (longer) than the former hand amplitude. That means performing hand movements while perceiving perturbed visual feedback originates after-effects in a subsequent movement. These findings speak in favor of a common representation of proximal and distal action effects, as proposed by the theory of event coding (Hommel et al., 2001). And furthermore (tool), actions are controlled with regard to their distal action effects. An increasing number of studies provides evidence for the dominance of distal action effects: for tool use (e.g., Kunde et al., 2007; Sutter, 2007; Massen and Prinz, 2008; Müsseler et al., 2008; Sutter et al., 2008, 2011; Lukas et al., 2010; Janczyk et al., 2012).

Studies by Sutter and colleagues (Sutter, 2007; Sutter et al., 2011) demonstrated for tools with different sensorimotor transformations the dominance of visual action effects in motor control. They varied amplitude and size of visual targets and found a log linear increase in movement times as a function of movement difficulty. The fit of the data with Fitts’ law (Fitts, 1954) held for cursor movements controlled by a motion-transforming tool – a touchpad that translates finger movement on the pad surface into cursor movement on the monitor. But more interestingly, Fitts’ law held in a comparable way for cursor movements produced by a force-driven tool (isometric joystick). Note that controlling an isometric joystick does not require any ballistic hand movement. The cursor movements result from finger force applied on the joystick alone. Consequently, the findings support the action effect account claiming that Fitts’ law holds for action effect movements (i.e., the cursor movements), irrespective of the sensorimotor transformation of a tool. Or in other words, what counts most in this situation is the representation of the distal action effect (i.e., the cursor movement toward the stimulus), not the proximal effect (i.e., the hand manipulating the tool). As predicted by the ideomotor principle (James, 1890; Greenwald, 1970; for recent overviews of empirical evidence see, e.g., Hommel et al., 2001; Nattkemper and Ziessler, 2004), any intentional act requires a goal, that is, some anticipatory representation of the intended action effect. The anticipation of these action effects may fulfill a generative function in motor control: actors select, initiate, and execute a movement by activating the anticipatory codes of the movement’s effects. These may be representations of body-related effects, like the proprioceptive/tactile feedback from the moving hand manipulating a tool (proximal effects). However, the intended action effects when using a tool are the movements of the effective part of the tool displayed on the monitor (distal effects). If both, proximal and distal action effects were equally important for controlling tool actions then any discrepancy between them would be a constant source of interference. The human information processing system apparently solves this conflict by favoring the distal action effects – as has been supported by an increasing body of research – while the proximal action effects are attenuated (e.g., Fourneret and Jeannerod, 1998; Knoblich and Kircher, 2004; Müsseler and Sutter, 2009; Wang et al., 2012). And it seems, that with increasing age distal action effects become even more influential in perception and action. This demonstrated a recent study by Wang et al. (2012): younger and older adults sat in front of a robot-arm and placed their dominant hand on a handle attached to the tip of the robot-arm. The hand of participants and the robot-arm were covered from view. The robot produced one of six pre-defined trajectories in the shape of an acute (γ = 45° or 63° or 81°) or an obtuse triangle (γ = 99° or 117° or 135°). All triangles were isosceles and had a constant horizontal base of 26 cm. Participants were instructed to follow the movement of the robot-arm with their hand on the handle and to monitor their hand movement very carefully. During the movement participants received no feedback (condition 1) or perturbed visual feedback on a display (condition 2). In the latter condition the cursor produced a static equilateral right-angled triangle with a horizontal base of 26 cm. The cursor movement was synchronized with the robot-arm’s movement. After the completion of the movement participants were asked to evaluate the shape of their hand trajectory (acute or obtuse) by giving a verbal response. The results showed that participants were more uncertain about the shape of their hand trajectory when perturbed visual feedback was presented than when no feedback was present. This effect was more pronounced in older adults than in younger. The authors conclude that perturbed visual feedback attenuates the perception of hand movements and that older adults are more susceptible to distal action effects than younger adults.

When looking at developmental changes in motor behavior several studies demonstrate an increase of the amount of visual control in goal-directed aiming for the elderly: for instance, Pratt et al. (1994) investigated the impact of practice on movement kinematics of younger and older adults in a rapid aiming task. Participants manipulated a handle to perform aiming movements with a cursor on a display. Target amplitude and target size were always the same. The amount of practice varied between 100 trials (exp. 1) and 200 trials (exp. 2). For younger adults they found a modification in movement kinematics as a function of practice (exp. 1): the amount of visually controlled fine adjustments decreased (distance and time of secondary submovement decreased), so that movements became more pre-programmed (distance and time of primary submovement increased). These changes in movement kinematics represent an optimization in motor control according to Meyer et al., 1988; for an overview of empirical evidence see Elliott et al., 2001). Older participants did not adjust their motor behavior in such a way and remained controlling movements mostly visually. Even an extension of the practice phase (exp. 2) did not show any further adjustments of motor behavior.

Seidler-Dobrin and Stelmach (1998) varied the amount of visual feedback (movement execution with vs. without visual feedback) and investigated its impact on movement kinematics. Younger and older participant manipulated a lever to perform constant aiming movements with a cursor on a display. In the first and third block (40 trials each) visual feedback of the cursor movement was omitted in 10 of the trials after movement onset. The second block was the practice block (100 trials) in which visual feedback was constantly available. When visual feedback was omitted in the pre-practice block (first block), the distance of the primary submovement decreased (and consequently the distance of the secondary submovement increased) and endpoint accuracy decreased for both age groups. In the post-practice block (third block) young participants had been able to optimize movement control in the practice block – so that the primary submovement generally lengthened, independently of whether the visual feedback was present or not. In contrast, movement control in older participant did not benefit from practice with visual feedback, but remained the same and continued to highly depend on visual feedback.

In this context the present paper aims to explore age-related changes in distal action effect control. Participants were seated in front of a display and a digitizer tablet. A cover screened the tablet and the participant’s hand, so that participants received proprioceptive/tactile feedback from their moving hand without seeing the hand itself. We presented two horizontally arranged target boxes on the display and asked participants to move the cursor (via pen on the digitizer tablet) several times per trial back and forth between the boxes. We varied the relationship between hand amplitude and cursor amplitude by introducing different gain factors (1:1.22; 1:2.44; 1:4.88), and we varied visual target size. Consequently, the pre-defined hand amplitude remained constant within a block, and the cursor amplitude and the visual target size randomly varied from trial to trial. After the completion of a trial, participants were asked to evaluate the length of their hand amplitude. The experimenter recorded the verbal response, after that the next trial appeared. In line with the previously mentioned studies (Pratt et al., 1994; Seidler-Dobrin and Stelmach, 1998) we held the pre-defined hand amplitude constant, so that participants were asked to produce the very same hand movement throughout a block. Yet, the perturbation of the visual feedback by introducing different gain factors led to varying cursor amplitudes and visual target sizes. This procedure is not trivial, since although there are a number of studies using Fitts’ law to evaluate human-computer interaction (e.g., MacKenzie, 1992; Armbrüster et al., 2004; Sutter, 2007), previous studies often varied both hand movements and cursor movements with regard to amplitude and target size.

To disentangle action effects, we varied only distal action effects and kept the proximal action effects constant (cf. Rieger et al., 2005). The ideomotor principle (James, 1890; Greenwald, 1970) holds that any intentional action is controlled by anticipatory representations of the intended action’s effect. These anticipations may be directed toward the goal of the hand’s actions: if intended body-related effects control tool actions then movement times should remain constant, as the pre-defined hand amplitude remains constant within a block. However, and more likely as an increasing number of studies demonstrates, actors represent (tool) actions in terms of their intended distal action effects. In the present study, these are the effects of the cursor’s movements on the display. Thus, we hypothesize that if task difficulty of cursor movements varies in terms of cursor amplitude and visual target size (Fitts, 1954 law) then hand movement times should increase as a function of task difficulty. We predict an increase of hand movement times with increasing cursor amplitude and decreasing visual target size, even though the pre-determined hand amplitude remains constant. This effect was recently found in our lab for a young population (Sutter et al., 2011, exp. 2), and should be replicated in this study.

Furthermore, the theory of event coding (Hommel et al., 2001) proposes a common representation of proximal and distal action effects, i.e., the event code. Consequently, both action effects may interact in action control and action perception: for instance, it has been found that if proximal and distal action effects do not correspond distal action effects predominated action control while proximal action effect were attenuated (e.g., Fourneret and Jeannerod, 1998; Knoblich and Kircher, 2004; Müsseler and Sutter, 2009; Wang et al., 2012). More important, there is also some crosstalk between proximal and distal action effects affecting action perception (e.g., Ladwig et al., 2012). Participants performed a discrete aiming movement and received perturbed visual feedback on the display. When asked to replicate the formerly performed hand amplitude (now without visual feedback), replicated amplitudes were longer (shorter) when the formerly seen cursor amplitude was longer (shorter) than the formerly performed hand amplitude. In the present study we asked participants to judge their hand amplitude by giving a verbal response instead of a motor response. In line with the findings of crosstalk between proximal and distal action effects we hypothesize that if distal action effects are predominant and therefore superpose proximal action effects then amplitude judgments of one’s own hand movements should be more affected by (varying) cursor amplitudes than by (constant) hand amplitudes. We predict an increase in amplitude judgments of one’s own hand movements with increasing cursor amplitude, although the pre-determined hand amplitude remains constant.

Finally, the present study investigated younger and middle-aged adults and expects age-related changes in distal action effect control. The optimized submovement model (Meyer et al., 1988) defines optimization of motor control when motor execution shifts over time on task from being more visually controlled to being more pre-programmed. Evidence has been presented above that older adults do not optimize movement execution in the same way as younger adults (e.g., Pratt et al., 1994; Seidler-Dobrin and Stelmach, 1998). Thus, while the amount of pre-programming increased in motor control of younger adults, older adults remained controlling movements visually. In line with these findings we hypothesize that middle-aged adults rely more on visual feedback than younger adults, and therefore the impact of distal action effects should more intensively unfold on hand movement times and judgments of hand amplitude of middle-aged participants than on the younger adults. We predict a stronger increase of hand movement times with increasing cursor amplitude and decreasing visual target size for middle-aged adults than for younger adults. For judgments of hand amplitude we also predict an interaction between age and cursor amplitude: for middle-aged adults we predict a stronger increase in amplitude judgments with increasing cursor amplitude than for younger adults.

## Materials and Methods

### Participants

Fifteen students from the RWTH Aachen University (seven female, 17–34 years of age, mean age 24 years) participated for pay or course credit. Another 15 adults (eight female, 55–67 years of age, mean age 59 years) followed a call in a local newspaper and participated for pay. Thirteen of fifteen younger adults were graduate students, one participant was a high school-student and one participant had a profession based on an apprenticeship. Thirteen of fifteen middle-aged participants had professions based on a university degree (equivalent to a master degree), two of them had professions based on an apprenticeship. All participants reported to use a computer and a computer mouse daily (younger adults: *M* = 2.7 h/days; SD = 2.2; middle-aged adults: 1.4 h/days; SD = 1.4; *F*(1, 25) = 3.12; *p* = 0.089; η^2^ = 0.11). All participants had normal or corrected-to-normal vision.

### Apparatus, task, and stimuli

Participants sat in front of a digitizer tablet (WACOM Intuos2 A3) that was operated with a pen (WACOM Intuos2 Grip Pen). Experimental tasks were presented on a 17′′ CRT display (EIZO F563-T) with a 1024 × 768 resolution. A cover screened the digitizer tablet and the moving hand from the view. On top of the cover a measuring tape was fixated. Participants were only able to see the display on which the cross-hair cursor (length 0.8 cm × 0.8 cm) and the target boxes were presented. The task involved moving the cursor back and forth between two horizontally arranged target boxes. Each trial lasted until 25 error-free movements occurred. This task design was adapted from Rieger et al. (2005). After finishing a trial participants were asked to estimate the average hand amplitude of the successful 25 movements.

The movement distance of the hand (hand amplitude) was the same for all trials within a block (20, 40, or 60 mm). Within each block movement distance of the cursor (cursor amplitude) varied as a result of gain factor [1.22 (low gain), 2.44 (middle gain), and 4.88 (high gain)]: the cursor amplitude was 24 (low gain), 48 (middle gain), and 97 mm (high gain) within the 20-mm block, 48, 97, and 195 mm within the 40-mm block, and 73, 146, and 292 mm within the 60-mm block. Additionally, within each block the target sizes varied randomly with 5, 10, 20, and 40 mm. The combination of 24-mm cursor amplitude and 40-mm target size in the 20-mm block was skipped from the procedure, as overlapping target boxes resulted.

### Procedure and design

Participants were instructed to continuously move the cursor back and forth between the two target boxes. As soon as they reached one target box the movement direction should be reversed without pausing in the target box. The instruction stressed the need to move continuously, and to move as fast and turn as accurately as possible. When 25 error-free movements were performed the screen turned blank. Participants then made a verbal judgment about the average length of their hand amplitude on the tablet (in cm), which was recorded by the experimenter.

The participants worked throughout the three blocks of hand amplitudes. The order of blocks was counterbalanced across participants. Within a block, cursor amplitude and visual target size were randomly varied. Each block consisted of 11 amplitude/size combinations (20-mm block) or 12 amplitude/size combinations (40- and 60-mm block) with 25 repetitions and additional 3 × 25 training trials in advance of the experimental trials. In total, the experiment lasted about 45 min.

## Results

For each block of hand amplitude data were separately analyzed. The *mean deviation* between the pre-determined hand amplitude and the estimated hand amplitude was analyzed with ANOVAs with the within-subject factors gain (low, middle, and high) and the between-subject factor age (young and middle-aged). Due to technical failure the verbal judgments of one middle-aged participant were not recorded. The *mean* *movement time* (the interval between a target-to-target movement, averaged across a successful 25 movement cycle) was calculated for error-free trials and analyzed with ANOVAs with the within-subject factors gain (low, middle, and high) and target size (5, 10, 20, and 40 mm), and the between-subject factor age (young and middle-aged). *Mean* e*rror rates* were calculated on the number of trials, where the reversal point of movement was outside the target box (averaged across a successful 25 movement cycle). A reversal point between start box and target box represents an undershoot, a reversal point behind the target box represents an overshoot. Before further analysis error rates were arc sin transformed. Then, data were analyzed with ANOVAs with the within-subject factors gain (low, middle, and high) and target size (5, 10, 20, and 40 mm), and the between-subject factor age (young and middle-aged). Additionally, for error trials we calculated the mean deviation between the pre-determined hand amplitude and the actual hand amplitude (=over-/undershoot). *Mean over-/undershoots* were analyzed with ANOVAs with the within-subject factors gain (low, middle, and high) and the between-subject factor age (young and middle-aged).

### Mean deviation of judgments

**Figure 1:**
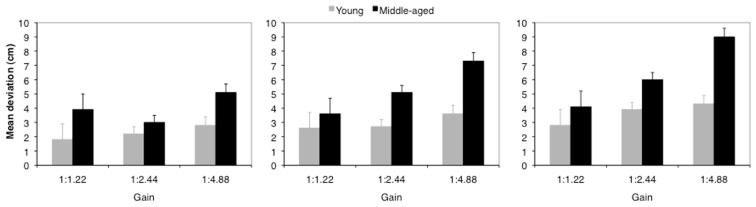
**Mean deviation (cm) between pre-determined hand amplitude and estimated hand amplitude for a pre-determined hand amplitude of 20 mm (left), 40 mm (middle), and 60 mm (right)**. Error bars represent the standard error of the mean.

Figure 1 depicts the results for the mean deviations of judgments.

For all hand amplitudes the analyses revealed a significant main effect for the factor age [20 mm: *F*(1, 27) = 4.10; *p* = 0.053; η^2^ = 0.13; 40 mm: *F*(1, 27) = 6.83; *p* = 0.014; η^2^ = 0.20 and 60 mm: *F*(1, 27) = 4.77; *p* = 0.038; η^2^ = 0.15]. The factor gain reached significance for the 40 mm and 60 mm amplitude, while for the 20 mm amplitude a corresponding trend was observed [20 mm: *F*(2, 54) = 2.81; *p* = 0.69; η^2^ = 0.09; 40 mm: *F*(2, 54) = 11.21; *p* < 0.001; η^2^ = 0.29; 60 mm: *F*(2, 54) = 7.46; *p* = 0.001; η^2^ = 0.22]. Furthermore, the factors age and gain interacted significantly for the 40 mm amplitude, a corresponding trend was observed for the 60 mm amplitude [20 mm: *p* = 0.364; 40 mm: *F*(2, 54) = 3.63; *p* = 0.033; η^2^ = 0.12; 60 mm: *F*(2, 54) = 2.44; *p* = 0.097; η^2^ = 0.08].

Across amplitudes middle-aged adults generally overestimated hand amplitudes stronger than younger adults (20 mm: 4.0 vs. 2.3 cm; 40 mm: 5.3 vs. 2.9 cm; and 60 mm: 6.4 vs. 3.7 cm). Concerning the impact of distal action effects results showed that although the pre-defined hand amplitude remained constant judgments increased as a function of gain (40 mm: by 2.4 cm and 60 mm: by 3.2 cm).

*Post hoc* tests with Bonferroni adjustments were carried out on judgments of younger and middle-aged adults separately. All judgments significantly deviated from zero (*p*s < 0.05). Considering the interaction between age and gain (40 mm hand amplitude) *post hoc* tests confirmed an increase in judgments as a function of gain for middle-aged adults (*p* < 0.05), but not for younger adults. Consequently, distal action effects – in terms of gain – mainly affected judgments of middle-aged adults (40 mm amplitude).

### Mean movement time

**Figure 2:**
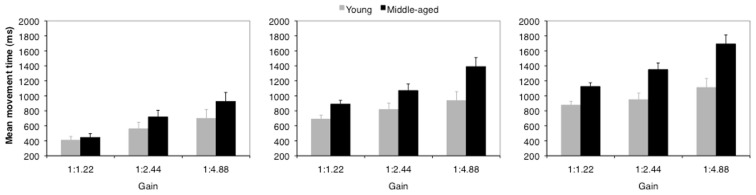
**Mean movement times of younger (gray) and middle-aged (black) adults as a function of gain for a hand amplitude of 20 mm (left), 40 mm (middle), and 60 mm (right)**. Error bars represent the standard error of the mean.

Mean movement times as a function of age and gain are depicted in Figure 2. For all hand amplitudes analyses showed a significant main effect for the factor gain [20 mm: *F*(2, 56) = 43.97; *p* < 0.001; η^2^ = 0.61; 40 mm: *F*(2, 56) = 31.68; *p* < 0.001; η^2^ = 0.53; 60 mm: *F*(2, 56) = 38.42; *p* < 0.001; η^2^ = 0.58]. The main effect of the factor age was significant for the 60 mm hand amplitude only [*F* (1, 28) = 4.03; *p* = 0.054; η^2^ = 0.13]. Analyses further revealed significant interactions between the factors age and gain for the 40 and 60 mm hand amplitudes [40 mm: *F*(2, 56) = 4.0; *p* = 0.024; η^2^ = 0.13; 60 mm: *F*(2, 56) = 6.51; *p* = 0.003; η^2^ = 0.19] and a corresponding trend for the 20 mm hand amplitude [20 mm: *F*(2, 56) = 2.73: *p* = 0.074; η^2^ = 0.09].

As depicted in Figure 2 movement times generally increased with a high gain compared to the low and middle gain. This is insofar remarkable, because hand movement times arising from constant hand amplitudes rose as a result of the increasing cursor amplitude. For longer hand amplitudes (40–60 mm) the impact of gain stronger affected middle-aged adults than younger adults.

The factor target size revealed significant main effects across all hand amplitudes [20 mm: *F*(3, 84) = 113.70; *p* < 0.001; η^2^ = 0.80; 40 mm: *F*(3, 84) = 97.47; *p* < 0.001; η^2^ = 0.78; 60 mm: *F*(3, 84) = 69.64; *p* < 0.001; η^2^ = 0.71]. Target size significantly interacted with age [20 mm: *F*(3, 84) = 3.73; *p* = 0.014; η^2^ = 0.12; 40 mm: *F*(3, 84) = 2.74; *p* = 0.048; η^2^ = 0.09; 60 mm: *F*(3, 84) = 6.10; *p* = 0.001; η^2^ = 0.18]. Finally, gain and target size interacted significantly for the 20 mm hand amplitude [*F*(6, 168) = 7.78; *p* < 0.001; η^2^ = 0.22]. Other main effects or interactions did not reach significance (*p*s > 0.222).

In all gain conditions movement times were lowest for the largest target and increased as a function of target size. Concerning the factors age and target size, movement times increased from 406 to 952 ms for younger adults and from 390 to 1304 ms for middle-aged adults for the 20 mm amplitude. For the 40 mm (60 mm) hand amplitudes movement times increased from 497 to 1260 ms (702 to 1363 ms) for younger adults and from 589 to 1724 ms (789 to 2171 ms) for middle-aged adults. This shows that target size had a stronger impact on movement times of middle-aged adults than on movement times of younger adults.

### Mean error rate and mean over-/undershoot in error trials

**Figure 3:**
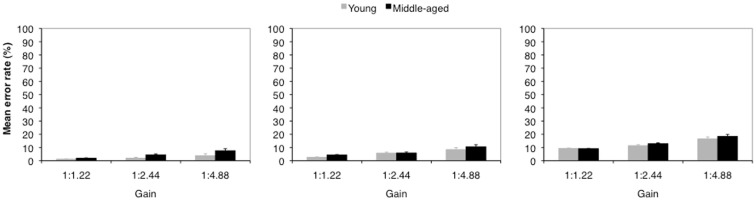
**Mean error percentages of younger (gray) and middle-aged (black) adults as a function of gain for a hand amplitude of 20 mm (left), 40 mm (middle), and 60 mm (right)**. Error bars represent the standard error of the mean.

Figure 3 depicts the mean error rate as a function of age and gain. Across all hand amplitudes a significant main effect of the factor gain was found [20 mm: *F*(2, 56) = 13.80; *p* < 0.001; η^2^ = 0.33; 40 mm: *F*(2, 56) = 24.68; *p* < 0.001; η^2^ = 0.47; 60 mm: *F*(2, 56) = 15.71; *p* < 0.001; η^2^ = 0.36]. The main effect of the factor age was significant for the 20 mm hand amplitude only [*F*(1, 28) = 5.84; *p* = 0.022; η^2^ = 0.17]. For the 20 mm hand amplitude error rates increased as a function of gain from 2 to 6% (young: *M* = 2%; middle-aged: *M* = 5%). For the 40 mm (60 mm) hand amplitude gain alone mediated error rates with a general increase from 4 to 9% (9–17%).

The factor target size revealed significant main effects for all hand amplitudes [20 mm: *F*(3, 84) = 43.67; *p* < 0.001; η^2^ = 0.61; 40 mm: *F*(3, 84) = 96.79; *p* < 0.001; η^2^ = 0.78; 60 mm: *F*(3, 84) = 66.05; *p* < 0.001; η^2^ = 0.70]. The interaction between gain and target size was significant for the 40 mm amplitude [*F*(6, 168) = 2.38; *p* = 0.031; η^2^ = 0.08]. A corresponding trend was found for the 20 mm amplitude [*F*(6, 168) = 1.92; *p* = 0.079; η^2^ = 0.06]. For the 60 mm hand amplitude a significant interaction between the factors age and target size was observed [*F*(3, 84) = 2.86; *p* = 0.041; η^2^ = 0.09]. Other main effects or interactions were not significant (*p*s > 0.121).

Error rates increased stronger as a function of target size in the high gain condition than in the low gain condition. The pattern of results in error rates resembles that of movement times, so that data were not confounded by a speed-accuracy trade-off.

Error trials were further analyzed with regard to the mean over-/undershoots. The ANOVAs with the within-subject factors gain (low, middle, and high) and the between-subject factor age (young and middle-aged) showed significant main effects of the factor gain for all hand amplitudes [20 mm: *F*(2, 56) = 29.89; *p* < 0.001; η^2^ = 0.52; 40 mm: *F*(2, 56) = 17.19; *p* < 0.001; η^2^ = 0.38; 60 mm: *F*(2, 56) = 18.80; *p* < 0.001; η^2^ = 0.40]. All other effects or interactions did not reach significance (*p*s > 0.134).

For the 20 mm hand amplitude with low gain we observed a small overshoot (+1.54 mm), for all other conditions participants undershot the target area by −2.52 mm (min.) to −16.56 mm (max.). Undershoots increased as a function of gain. That means, although actual hand amplitudes in error trials deviated from the pre-defined hand amplitude, the observed undershoots can not account for overestimations observed in hand amplitude judgments.

## General Discussion

In this study we investigated the susceptibility of older adults to distal action effects. Younger and older participants performed a Fitts’ task on a digitizer tablet without seeing their hand and the tablet directly. Visual feedback was presented on a display in that way, that cursor amplitude and visual target size varied while the pre-defined hand amplitude remained constant. In accordance with distal action effects being predominant in controlling tool actions (e.g., Kunde et al., 2007; Sutter, 2007; Massen and Prinz, 2008; Müsseler et al., 2008; Sutter et al., 2008, 2011; Lukas et al., 2010; Janczyk et al., 2012) we proposed an increase in hand movement times as a function of cursor amplitude and visual target size (Fitts, 1954). Since pre-defined hand amplitudes remained constant, any changes in distal action effects should be mirrored by variations in movement times if actions were predominantly distally controlled. And indeed, this is what we found. Hand movement times were strongly determined by cursor amplitude and target size. Both visual task characteristics accounted for most variance in movement times (η^2^ between 0.53 and 0.80). This strong impact of distal action effects on tool actions is a successful replication of a recent finding (Sutter et al., 2011, exp. 2). And to further this, a similar and even stronger influence of visual task characteristics on movement times was found for middle-aged adults. This result was being hypothesized on the fact, that the elderly control manual actions to a larger amount visually than the younger (e.g., Haaland et al., 1993; Pratt et al., 1994; Seidler-Dobrin and Stelmach, 1998). To rule out that any speed-accuracy trade-off may have mediated the age effect, we looked at error rates in detail: across age groups we found that error rates were strongly moderated by gain and visual target size. However, they followed the same pattern of results as found in movement times: both dependent variables rose due to extensions in gain and to reductions in visual target size. Between age groups error rates only differed significantly for the 20 mm hand amplitude. In that case error rates were 3% higher for middle-aged adults than for younger adults (5 vs. 2%). But, at the same time middle-aged adults were also slower than younger adults. Thus, the often found strategic differences (cf. Pachella, 1974; Welford, 1976) between younger adults – emphasizing speed and neglecting accuracy – and older adults – emphasizing accuracy and neglecting speed – could not account for the age differences found in this experiment. Thus, we can preclude speed-accuracy trade-off as confounding factor.

However, another possible confound may emanate from the methodology we used. The study was designed as a cross-sectional study. We controlled age groups for specific demographic factors, like gender and daily computer usage. The latter factor might be critical for the present experiment: we observed a trend for middle-aged adults spending less time per day in front of a computer compared to younger adults. The relationship between computer mouse movements and cursor movements is basically similar to the transformation scaling gain used in the present experiment. So it could be that less computer usage led to a disadvantage for the middle-aged and that this accounted for the age differences found in the present experiment.

However, on the one hand, we asked participants about their daily computer usage, but not about their exposure to computers and computer input devices over their life span. This should be done in future studies, because it seems to be very likely, but we can only speculate about this point at the moment, that middle-aged adults spend more years (but less time per day) using a computer, and vice versa for younger adults. Consequently, the amount of life time exposure might even the amount of daily usage out. On the other hand, it is known that transformations scaling gain are very easy to learn and adaptation occurs very fast (Bedford, 1994; Bock and Burghoff, 1997; Seidler et al., 2001; Rieger et al., 2005; Sutter et al., 2008). Moreover, middle-aged and younger adults comparably improve performance by practice (e.g., Armbrüster et al., 2007) and adapt to gain transformations (e.g., Heuer and Hegele, 2007). Thus, although we can not fully rule out a possible confound, previous studies give reason that the group differences found in our cross-sectional study relate to developmental changes. However, further light could be shed on this point by conducting a longitudinal study.

Our second hypothesis was concerned with the impact of distal action effects on the proprioceptive/tactile perception. There is evidence for actors being less aware of what they do with their hands when there is a discrepancy between proximal and distal action effects (e.g., Fourneret and Jeannerod, 1998; Knoblich and Kircher, 2004; Müsseler and Sutter, 2009; Wang et al., 2012). In the experiment we asked participants to estimate the average amplitude of their hand movement after finishing 25 error-free movements. In general, judgments were most precise when the low gain only slightly perturbed the relation between hand and cursor amplitude and it became increasingly imprecise with higher gain factors. That means, although the pre-determined hand amplitude remained constant, participants were not really aware of what they were doing with their hand. Moreover, middle-aged participants stronger overvalued their hand amplitude than younger participants. This was particularly the case for longer hand amplitudes.

A critical point is, that error rates increased with increasing gain and longer hand amplitudes, too. It could have been that participants systematically overshot the target area in error trials and that this biased their judgments. Fortunately, the data revealed a contrary pattern of results, and confirms distal action effects mediated the perceptual bias.

To shed further light on these findings age-related changes in sensory performance will be discussed in more detail. It is well known that proprioceptive acuity declines with age (e.g., Cooke et al., 1989; Darling et al., 1989; Adamo et al., 2007; Boisgontier et al., 2012). For instance, Adamo et al. (2007) compared the proprioceptive acuity between younger and older participants in an elbow-position matching task. Having only proprioceptive information to match a former position increased matching errors for the older adults more intensely than for younger. Concerning the present study, judgment errors resembles this pattern of results. Although participants were instructed to monitor their hand movement carefully, judgments were quite inaccurate across all conditions. Judgment errors were more pronounced in middle-aged adults than in younger adults, and inaccuracy more strongly increased in middle-aged adults when cursor amplitude increased. The same impact of visual feedback was also found in motor behavior. Thus, middle-aged adults obviously rely on visual feedback in perceiving and controlling actions. One could argue that the tendency of older adults to allocate more resources on controlling movements visually (see also Haaland et al., 1993) might be a useful (compensation) mechanism against increasing neural noise in the motor system (Welford, 1984). Quantitative models of multisensory integration (Ernst and Banks, 2002) assume that information from all involved senses contribute to a percept in an optimized fashion, so that the reliability of the percept is maximized. Concerning motor actions, the proprioceptive information of limb movements, however, is highly variable (e.g., Cooke et al., 1989; Darling et al., 1989; Fourneret and Jeannerod, 1998; Knoblich and Kircher, 2004; Adamo et al., 2007; Müsseler and Sutter, 2009; Boisgontier et al., 2012; Wang et al., 2012), and less reliable than the visual perception (e.g., van Beers et al., 1998). Aging, however, increases the variance in the proprioceptive sense (Welford, 1984). And consequently, it makes perfect sense that the visual information becomes even more important with increasing age. Further experiments are needed to substantiate this interpretation. One way to investigate the integration of visual and proprioceptive information is to add noise to either the visual or the haptic sense (e.g., Serwe et al., 2009; Sutter and Ladwig, 2012). For instance applying vibration to a moving hand adds noise to the afferent information of the proprioceptive sense. Consequently, actions that were controlled by their proximal action effects when vibration was not present shifted to be visually controlled when the vibration was in effect (Sutter and Ladwig, 2012).

In conclusion, based on the cognitive account of action effect control (James, 1890; Greenwald, 1970; Hommel et al., 2001) our results demonstrated that distal action effects predominantly determined how actors perceive and interact with tools. Movement times varied as a function of gain while the pre-determined hand amplitude remained constant. This is insofar surprising, as it represents a highly demanding and resource-limiting behavior. If participants had been able to ignore the visual feedback completely, then the very same motor program (e.g., Schmidt, 1988; Elliott et al., 2001) would have fitted for all movements within a block. Whereas the younger participants (according to their judgments) seemed to have realized that they were performing the same movements all the time, movement times nevertheless increased due to the cursor amplitude. The influence of the visual feedback unfolded even more intensely in middle-aged adults. On the one hand age-related limits in cognitive processing capacities can be assumed, since increasing task difficulty extended the performance gap between younger and middle-aged adults. On the other hand and more likely, age-related changes in the proprioceptive acuity play the crucial role in this context. Thus, design and application of tools for the elderly should account for this.

## Conflict of Interest Statement

The authors declare that the research was conducted in the absence of any commercial or financial relationships that could be construed as a potential conflict of interest.

## References

[B1] Adamo D. E. Martin B. J. Brown S. H. (2007). Age-related differences in upper limb proprioceptive acuity. Percept. Mot. Skills 104, 1297–1309 10.2466/pms.104.4.1297-1309 17879664

[B2] Armbrüster C. Sutter C. Ziefle M. (2004). “Target size and distance: important factors for designing user interfaces for middle-aged notebook users,” in Work with Computing Systems, eds Khalid H. M. Helander M. G. Yeo A. W. (Kuala Lumpur: Damai Sciences), 454–459

[B3] Armbrüster C. Sutter C. Ziefle M. (2007). Notebook input devices put to the age test: the usability of track point and touchpad for middle-aged adults. Ergonomics 50, 426–445 10.1080/00140130601127885 17536778

[B4] Bedford F. L. (1994). Of computer mice and men. Cah. Psychol. Cognit. 13, 405–426

[B5] Bock O. Burghoff M. (1997). Visuo-motor adaptation: evidence for a distributed amplitude control system. Behav. Brain Res. 89, 267–273 10.1016/S0166-4328(97)00069-7 9475634

[B6] Boisgontier M. P. Olivier I. Chenu O. Nougier V. (2012). Presbypropria: the effects of physiological ageing on proprioceptive control. Age 34, 1179–1194 10.1007/s11357-011-9300-y 21850402 PMC3448996

[B7] Cooke J. D. Brown S. H. Cunningham D. A. (1989). Kinematics of arm movements in elderly humans. Neurobiol. Aging 10, 159–165 10.1016/0197-4580(89)90025-0 2725811

[B8] Darling W. G. Cooke J. D. Brown S. H. (1989). Control of simple arm movements in elderly humans. Neurobiol. Aging 10, 149–157 10.1016/0197-4580(89)90024-9 2725810

[B9] Elliott D. Helsen F. Chua R. (2001). A century later: Woodworth’s (1899) Two-Component Model for goal-directed aiming. Psychol. Bull. 127, 342–357 10.1037/0033-2909.127.3.342 11393300

[B10] Ernst M. O. Banks M. S. (2002). Humans integrate visual and haptic information in a statistically optimal fashion. Nature 415, 429–433 10.1038/415429a 11807554

[B11] Fitts P. M. (1954). The information capacity of the human motor system in controlling the amplitude of movement. J. Exp. Psychol. 47, 381–391 10.1037/h0055392 13174710

[B12] Fourneret P. Jeannerod M. (1998). Limited conscious monitoring of motor performance in normal subjects. Neuropsychologia 36, 1133–1140 10.1016/S0028-3932(98)00006-2 9842759

[B13] Greenwald A. G. (1970). Sensory feedback mechanisms in performance control: with special reference to the ideo-motor mechanism. Psychol. Rev. 77, 73–99 10.1037/h0028689 5454129

[B14] Haaland K. Y. Harrington D. L. Grice J. W. (1993). Effects of aging on planning and implementing arm movements. Psychol. Aging 8, 617–632 10.1037/0882-7974.8.4.617 8292290

[B15] Heuer H. Hegele M. (2007). Learning visuo-motor gains at early and late working age. Ergonomics 50, 979–1003 10.1080/00140130701240828 17510818

[B16] Heuer H. Hegele M. (2009). Adjustment to a complex visuo-motor transformation at early and late working age. Ergonomics 52, 1039–1054 10.1080/00140130902912795 19626502

[B17] Hommel B. Müsseler J. Aschersleben G. Prinz W. (2001). The theory of event coding (TEC): a framework for perception and action. Behav. Brain Sci. 24, 869–937 10.1017/S0140525X01000103 12239891

[B18] James W. (1890). The Principles of Psychology. New York, NY: Dover

[B19] Janczyk M. Pfister R. Kunde W. (2012). On the persistence of tool-based compatibility effects. Z. Psychol. 220, 16–22 10.1027/2151-2604/a000086

[B20] Knoblich G. Kircher T. T. J. (2004). Deceiving oneself about being in control: conscious detection of changes in visuomotor coupling. J. Exp. Psychol. Hum. Percept. Perform. 30, 657–666 10.1037/0096-1523.30.4.657 15301616

[B21] Kunde W. Müsseler J. Heuer H. (2007). Spatial compatibility effects with tool use. Hum. Factors 49, 661–670 10.1518/001872007X215737 17702217

[B22] Ladwig S. Sutter C. Müsseler J. (2012). Crosstalk between proximal and distal action effects when using a tool. Z. Psychol. 220, 10–15 10.1027/2151-2604/a000085

[B23] Lukas S. Brau H. Koch I. (2010). Anticipatory movement compatibility for virtual reality interaction devices. Behav. Inform. Technol. 29, 165–174 10.1080/01449290902765191

[B24] MacKenzie I. S. (1992). Fitts law as a research and design tool in human-computer interaction. Hum. Comput. Interact. 7, 91–139 10.1207/s15327051hci0701_3

[B25] Massen C. Prinz W. (2008). Programming tool-use actions. J. Exp. Psychol. Hum. Percept. Perform. 33, 692–704 17563230 10.1037/0096-1523.33.3.692

[B26] Meyer D. E. Abrams R. A. Kornblum S. Wright C. E. Smith P. E. K. (1988). Optimality in human motor performance: ideal control of rapid aimed movements. Psychol. Rev. 95, 340–370 10.1037/0033-295X.95.2.183 3406245

[B27] Müsseler J. Kunde W. Gausepohl D. Heuer H. (2008). Does a tool eliminate spatial compatibility effects? Eur. J. Cogn. Psychol. 20, 211–231 10.1080/09541440701275815

[B28] Müsseler J. Sutter C. (2009). Perceiving one’s own movements when using a tool. Conscious. Cogn. 18, 359–365 10.1016/j.concog.2009.02.004 19289291

[B29] Nattkemper D. Ziessler M. (2004). Cognitive control of action: the role of action effects. Psychol. Res. 68, 71–73 10.1007/s00426-003-0145-6 15071742

[B30] Pachella R. (1974). “The interpretation of time in information-processing research,” in Human Information Processing: Tutorials in Performance and Cognition, ed. Kantowitz B. (Hillsdale: John Wiley & Sons), 41–80

[B31] Pratt J. Chasteen A. L. Abrams R. A. (1994). Rapid aimed limb movements: age differences and practice effects in component submovements. Psychol. Aging 9, 325–334 10.1037/0882-7974.9.2.325 8054180

[B32] Rieger M. Knoblich G. Prinz W. (2005). Compensation for and adaptation to changes in the environment. Exp. Brain Res. 163, 487–502 10.1007/s00221-004-2203-8 15742199

[B33] Schmidt R. A. (1988). Motor Control and Learning: A Behavioral Emphasis. Champaign, IL: Human Kinetics Publishers

[B34] Seidler R. D. Bloomber J. J. Stelmach G. E. (2001). Patterns of transfer of adaptation among body segments. Behav. Brain Res. 122, 145–157 10.1016/S0166-4328(01)00183-8 11334645

[B35] Seidler-Dobrin R. D. Stelmach G. E. (1998). Persistence in visual feedback control by the elderly. Exp. Brain Res. 119, 467–474 10.1007/s002210050362 9588781

[B36] Serwe S. Drewing K. Trommershäuser J. (2009). Combination of noisy directional visual and proprioceptive information. J. Vis. 9, 1–14 10.1167/9.13.1 19757906

[B37] Sülzenbrück S. Heuer H. (2012). Enhanced mechanical transparency during practice impedes open-loop control of a complex tool. Exp. Brain Res. 218, 283–294 10.1007/s00221-012-3011-1 22278111

[B38] Sutter C. (2007). Sensumotor transformation of input devices and the impact on practice and task difficulty. Ergonomics 50, 1999–2016 10.1080/00140130701510147 18033612

[B39] Sutter C. Ladwig S. (2012). Mirrored visual feedback limits distal effect anticipation. Exp. Brain Res. 218, 247–258 10.1007/s00221-012-3076-x 22331170

[B40] Sutter C. Müsseler J. Bardos L. (2011). Effects of sensorimotor transformations with graphical input devices. Behav. Inform. Technol. 30, 415–424 10.1080/01449291003660349

[B41] Sutter C. Müsseler J. Bardos L. Ballagas R. Borchers J. (2008). “The impact of gain change on perceiving one’s own actions,” in Mensch & Computer 2008, eds Herczeg M. Kindsmüller M. C. (München: Oldenbourg Wissenschaftsverlag), 147–156

[B42] Sutter C. Sülzenbrück S. (2012). Perceiving transformed movements when using tools. Exp. Brain Res. 218, 163–167 10.1007/s00221-012-3018-7 22456942

[B43] Sutter C. Sülzenbrück S. Rieger M. Müsseler J. (in press). Limitations of distal effect anticipation when using tools. New Ideas Psychol.

[B44] van Beers R. J. Sittig A. C. Denier van der Gon J. J. (1998). The precision of proprioceptive position sense. Exp. Brain Res. 122, 367–377 10.1007/s002210050525 9827856

[B45] Wang L. Sutter C. Müsseler J. Dangel R. J. Z. Disselhorst-Klug C. (2012). Perceiving one‘s own limb movements with conflicting sensory feedback: the role of mode of movement control and age. Front. Psychol. 3:289 10.3389/fpsyg.2012.00289 22908005 PMC3414862

[B46] Welford A. T. (1976). Motivation, capacity, learning and age. Int. J. Aging Hum. Dev. 7, 189–199 10.2190/9477-XQE6-Q52U-758J 1002324

[B47] Welford A. T. (1984). Psychomotor performance. Annu. Rev. Gerontol. Geriatr. 4, 237–273 6443552

